# Probiotics encapsulated gastroprotective cross‐linked microgels: Enhanced viability under stressed conditions with dried apple carrier

**DOI:** 10.1002/fsn3.3116

**Published:** 2022-11-04

**Authors:** Muhammad Afzaal, Farhan Saeed, Huda Ateeq, Muhammad Nadeem Akhtar, Ali Imran, Aftab Ahmed, Muhammad Aamir, Fakhar Islam, Iqra Yasmin, Yasir Abbas Shah, Muzzamal Hussain, Adnan Hameed, Roshan Kumar, Chinaza Godswill Awuchi

**Affiliations:** ^1^ Department of Food Sciences Government College University Faisalabad Faisalabad Pakistan; ^2^ University Institute of Diet and Nutritional Sciences The University of Lahore Lahore Pakistan; ^3^ Institute of Home Sciences University of Agriculture Faisalabad Pakistan; ^4^ Barani Agricultural Research Institute Chakwal Pakistan; ^5^ Department of Pharmacology Dev Bhoomi Institute of Pharmacy and Research Dehradun India; ^6^ School of Natural and Applied Sciences Kampala International University Kampala Uganda

**Keywords:** antacid, apple snack, encapsulation, gastroprotective microgels, probiotics, viability

## Abstract

In the current study, *Lactobacillus acidophilus* was encapsulated in sodium alginate and whey protein isolate, with the addition of antacids CaCO_3_ or Mg(OH)_2_. The obtained microgels were observed by scanning electron microscopy. Encapsulated and free probiotics were subjected to vitality assay under stressed conditions. Furthermore, dried apple snack was evaluated as a carrier for probiotics for 28 days. A significant (*p* ≤ .05) effect of antacid with an encapsulating agent was observed under different stressed conditions. During exposure to simulated gastrointestinal conditions, there were observations of 1.24 log CFU and 2.17 log CFU, with corresponding 0.93 log CFU and 2.63 log CFU decrease in the case of SA + CaCO_3_ and WPI + CaCO_3_ respectively. Likewise, high viability was observed under thermal and refrigerated conditions for probiotics encapsulated with SA + CaCO_3_. In conclusion, the results indicated that alginate microgels with CaCO_3_ are effective in prolonging the viability of probiotics under stressed conditions.

## Graphical abstract

The addition of antacids (CaCO_3_ or Mg (OH)_2_) has a key role in protecting encapsulated probiotics under stressed conditions. The production of dried apple snacks with encapsulated probiotics is appealing for improving the human health. Microencapsulation with gastro protective is a versatile technique for the production of functional foods.

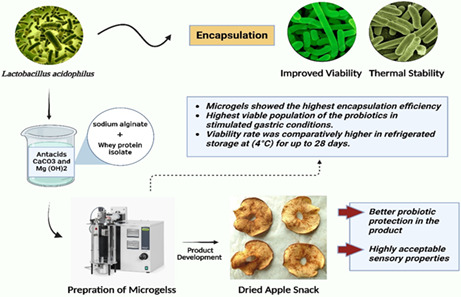

## 1 INTRODUCTION

Probiotics can influence and enhance the microbiota of the host (Omar et al., 2013), by boosting immunity and inhibit the pathogens to grow (Tripathi & Giri, 2014). Thus, they provide abundant health‐promoting benefits that includes improving overall immunity, lowering serum cholesterol, and improving lactose intolerance (Agrawal, 2005). To obtain the desired results, probiotic foods must contain at least 10^6^–10^7^ CFU/g live cells at the moment of ingestion (Terpou et al., 2019). Numerous factors, such as oxidative stress, drying process, and storage temperature, can have an impact on probiotic viability throughout food preparation and storage stages (Fiocco et al., 2020). Furthermore, the majority of the probiotics should remain intact during their transit through the gastrointestinal path until they reach the specific sites where they exhibit therapeutic effects (Doherty et al., 2012; Iqbal et al., 2021).

Microencapsulation has received a great deal of study to present (Chen et al., 2017). Probiotic microencapsulation not only protects the probiotic cells from harsh exterior environment but also allows the cells for their controlled release at specific places (Awuchi et al., 2022; Kia et al., 2018). Evidence has suggested that the survival chances of microencapsulated or un‐encapsulated probiotic cells during simulated gastric and intestinal circumstances depend on the strain (Oguntoye et al., 2021). Encapsulation methods employing macro, micro, and nano materias have been proven to be of great significance in improving probiotic utilization (Islam, Noman, et al., 2022; Islam, Saeed, et al., 2022; Morya et al., 2022). In addition, the shielding effect varies with different wall materials, microencapsulation techniques, and artificial intestinal and gastric conditions (Anitha & Sellamuthu, 2021; Rafiq et al., 2022; Xing et al., 2015). More systematic research is needed to offer more information regarding the protective effects of microencapsulation on probiotics.

*Bifidobacterium longum* entrapped in alginate hydro beads with chitosan layer showed an enhanced survivability after transit through simulated digestive conditions (Yeung et al., 2016). However, limited studies have shown that colloidal delivery methods may reliably sustain the viability of probiotics even after exposure of 2 h under gastric environment (Yeung et al., 2016). The major purpose behind the poor gastric viability of microgel encapsulated probiotics is due to the inactivated bacteria in which hydrogen ions were diffused through the hydrogel network. Zheng et al. (2017) prepared alginate microbeads that contained antacid Mg(OH)_2_, which could neutralize hydrogen ions and therefore maintain a neutral internal pH even when the microgels were inoculated in gastric conditions. These antacid‐loaded microgels are therefore useful for enhancing the probiotic cell viability through oral route (Yao et al., 2017).

In the current study, probiotic bacterial strain, *Lactobacillus acidophilus*, was encapsulated in sodium alginate and whey protein isolate microgels containing two different types of antacids, CaCO_3,_ and Mg (OH)_2_. Afterward, the encapsulated microgels were added to dried apple snacks and observed for probiotic viability. The obtained results from this study provided valuable material for improving the performance of probiotic‐loaded delivery systems for various applications in functional foods and beverages.

## 2 MATERIALS AND METHODS

### 2.1 Experimental study

The glassware was purchased from Thermofisher, USA. Encapsulating materials and chemicals were purchased from Merck, USA. The freeze‐dried culture of *L. acidophilus (*ATTCC 8826) was obtained from the National Institute of Food Science & Technology (NIFSAT), University of Agriculture Faisalabad, Pakistan. Apples (variety Kala Kulu) were purchased from Jhang bazar market of Faisalabad (Pakistan). The laboratory equipments were available in different laboratories of the Department of Food Sciences, Government College University Faisalabad, and were used for research purposes.

### 2.2 Activation of bacterial culture

Activation of freeze‐dried cell culture was done by following the method of Afzaal, Saeed, Ateeq, et al. (2020); Afzaal, Saeed, Hussain, et al. (2020); Afzaal, Saeed, Saeed, et al. (2020), with slight modifications. Solution of Man, Rogosa, and Sharpe agar (M.R.S agar, LAB093; Lab M Limited) was prepared by adding 70 g of agar in 1 L distilled water. The media was dissolved and autoclaved and plates were prepared for propagation of *L. acidophilus* (ATTCC 8826). Bacterial culture was incubated in an anaerobic environment at 37°C for 24 h using an incubator (BC‐5501; Memmert). Afterward, the obtained cells were centrifuged (750286 EA; Thermo Fisher Scientific Inc.) at 4000 rpm at 4°C for 10–15 min and the media was decanted. Cells were again suspended in freshly made MRS media and incubation (37°C) was done for an additional 20 h. The cells were harvested, weighed in, and data were recorded. The cell concentration was adjusted at 10^10^ CFU/ml.

### 2.3 Probiotic microencapsulation

#### 2.3.1 Whey protein isolate microgels preparation

Cells obtained by centrifugation (4000 rpm for 10–15 min) and washed thoroughly using sterilize peptone (15 ml) and afterward washed with 22 ml of aseptic distilled water. Probiotic microgels containing antacids were prepared by mixing concentrated *L. acidophilus* cells (5 ml) with 30 g whey protein isolate (WPI) powder in the presence of an antacid (CaCO_3_ or Mg (OH)_2_) (2:2, v/v) (Mehra et al., 2021; Wang et al., 2022). A volume of 125 ml sunflower oil was added to the WPI solution, and both the antacids CaCO_3_ and Mg (OH)_2_ (2:2, v/v) were added in the solution separately. Afterward, it was subjected to the preparation of microgels using an encapsulator (Büchi B‐3910 Encapsulator). Microencapsulation of *L. acidophilus* cells was performed as described by Wang et al. (2022) and Mehra et al. (2021), with minor modifications. An injection nozzle having 180–200 μm diameter was and following operating conditions were followed: vibration frequency = 750 Hz, driving pressure = 500 mbar and electrode potential = 750 V. The samples were collected in a 10% (w:v) calcium chloride solution for microbead formation.

#### 2.3.2 Preparation of alginate microgels

Alginate microgels were prepared by adopting the injection‐gelation method (Zhang, 2020) with some minor amendments. For encapsulation, the cells obtained by centrifugation were washed thoroughly with sterile peptone water (15 ml) and then rewashed twice with 22 ml of distilled water. Microbeads were formed by mixing 5 ml of *L. acidophilus* cell suspension in 2% w/v sodium alginate solution (200 ml) and both the antacids CaCO_3_ and Mg (OH)_2_ at a ratio of 2:2 v/v were added separately. Afterward, buffer solution of phosphate (pH 7) was added dropwise in order to adjust the final pH of the solution and stirred for 60 min at 50°C. Furthermore, the temperature was lowered to 35°C with constant stirring till a uniform solution was obtained. Microgels were obtained by injecting the mixture through a nozzle (180–200 μm diameter) using an encapsulator (Büchi B‐3910 Encapsulator; Flawil). The conditions used to obtain microgels were: 750 Hz, driving pressure = 500 mbar and electrode potential = 750 V. Antacid‐loaded microgels were then held in the calcium solution (0.05 ml) for 15 min at room temperature before being removed to promote hardening of gels. The obtained microgels were filtered and washed twice using sterilized distilled water.

After the preparation of beads from both types of antacids and encapsulating materials, the treatments were given the names, SA + Mg (OH)_2_ and SA + CaCO_3_ beads prepared from sodium alginate with both antacids. WPI + Mg (OH)_2_ and WPI + CaCO_3_ that were formed from Whey Protein Isolate with both antacids (Mg (OH)_2_ and CaCO_3_). Free cells of *L. acidophilus* were given the name F_c_.

### 2.4 Characterization of encapsulated whey protein isolate and alginate microgels

#### 2.4.1 Particle size determination

The particle size of gastroprotective microbeads was examined immediately following microencapsulation using a compound microscope (Mastersizer S; Malvern Instruments), to make sure that beads were of the correct size and shape.

#### 2.4.2 Scanning electron microscopy (SEM)

A high‐resolution scanning electron microscope (Cube series‐. Emcraft South Korea) available at the physics department‐GCUF was used to collect the micrograph. The prepared microbeads were subjected to structural morphology determination as described by Yao et al. (2017) with slight modification.

### 2.5 Encapsulation efficiency

The encapsulation efficiency was calculated by adopting the method of Afzaal, Saeed, Ateeq, et al. (2020). The microbeads beads of sodium alginate and whey protein isolate loaded with antacids Mg(OH)_2_ or CaCO_3_ were taken randomly, and disintegration of the beads was done using a stomacher with the pour plate technique, the number of cells released was measured. The findings were expressed as units/bead (CFU/bead) forming several colonies. Using the following formula, the importance of encapsulation efficiency was evaluated:

$$\text{Encapsulation Efficiency}=\mathrm{EE}=\left(\frac{{\mathrm{Log}}_{10}N}{{\mathrm{Log}}_{10}N_{0}}\right)\times 100$$

### 2.6 Survival of un‐encapsulated and encapsulated probiotics in gastrointestinal fluids

Using the approach as defined by Gu et al. (2019), free and encapsulated (sodium alginate and whey protein isolate) microgels were evaluated in simulated gastrointestinal conditions. Particularly, simulated gastric fluid (SGF) was prepared with the addition of sodium chloride (2 g), 6 M hydrochloric acid (7 ml) into 1000 ml distilled water and sterilized to ensure aseptic conditions. Simulated intestinal juice (SIJ) was prepared by dissolving sodium chloride (3.75 M) and calcium chloride (0.25 M) in phosphate buffer (pH 7). Prepared simulated solutions were subjected to autoclave for an aseptic environment before the experiment. Free and encapsulated microgels were consecutively added to SGJ and SIF for 120 min. The survival of unencapsulated and encapsulated probiotics was reported over a time interval of 0, 30, 60, 90, and 120 min. The final readings were noted.

### 2.7 Analysis of free and encapsulated microgels under heat treatment

The feasibility of *L. acidophilus* encapsulated microgels and free probiotics was determined by subjecting them to elevated temperature following the procedure of Fang and Bhandari (2012) with some minor amendments. Free cell and encapsulated cells of *L. acidophilus* (10^10^ CFU) were inoculated in test tubes having 9 ml saline solution (1% w/v). Additionally, the test tubes were incubated for 10 min in water bath at 63°, 65°, and 72°C. Subsequently after incubation, the test tubes were cooled down to normal room temperature (∼25°C). The viability of the unencapsulated and encapsulated microgels of *L. acidophilus* was then assessed by spread plate method using MRS agar as a growth medium at 37°C in an incubator (BC‐5501; Memmert) for 24 h.

### 2.8 Viability of free and encapsulated microgels during refrigeration storage

For the analysis of probiotic resistance against refrigeration temperature, the process of Lemos Junior et al. (2020) and Terpou et al. (2019) were followed with slight modification. The viability of *L. acidophilus* under refrigeration storage was evaluated by incubating 0.4 ml (approximately 9.5 log CFU/g) of free and encapsulated cells in 1.8 ml of germ‐free sodium chloride solution (0.5%, w/v) and kept in the refrigerator at 4°C for 28 days.

### 2.9 Product development

Dried apple snacks were prepared by adopting the technique as previously reported by Afzaal, Saeed, Ateeq, et al. (2020); Afzaal, Saeed, Hussain, et al. (2020); Afzaal, Saeed, Saeed, et al. (2020). First, apples were washed using normal tap water ensuring that dirt particles were removed. Peeling was done using a peeler and apples were sliced (diameter 9 mm, width 6 mm). Afterward, apples were blanched using water bath at 75°C for 2–3 min to prevent apples from enzymatic browning.

*Lactobacillus acidophilus* as a probiotic in both free and encapsulated form, ranging from 9.5–10 log CFU/g were added in sterile peptone water (∼1%), and apple slices were immersed in an aqueous solution (2:4 apple/solution ratio w/v) for 10 min at a room temperature and with constant stirring. Furthermore, apples in peptone water solution were left in a controlled environment for 10–15 min ensuring proper probiotics attachment on apple surface. The apples were then separated from the solution and were dried in a conventional oven (Westpoint Oven‐WF‐4800) at 38–40°C for a maximum of 40–50 min. The dried apple snack obtained was cooled down to ambient room temperature (∼25°C) in a desiccator for 20 min and afterward, stored in airtight food grade packages for further storage. Dried apple snack was stored for 28 days of storage study at 4°C in food graded storage bags and analysis was conducted at an interval of 7 days. The treatments for dried apple snack were named as “AS (SA + Mg (OH)_2_)” and “AS (SA + CaCO_3_)” dried apple snack having sodium alginate capsules with antacids. The dried apple snack encapsulated with whey protein isolate were given the names as “AS (WPI + Mg (OH)_2_)” and “AS (WPI + CaCO_3_).” However, a controlled sample of dried apple snack was quoted as “AS” and dried apple snack with free cells of *L. acidophilus* was named as “AS_FC_.”

### 2.10 Determination of pH of apple snack

The pH of apple snacks was determined by a digital pH meter following AOAC (2009). Apple snack was immersed in peptone water and mixed well before determining the pH. Readings were noted as a mean of three replicates.

### 2.11 Probiotic enumeration

The viability of probiotics in dried apple snack treatments was determined as described by Nualkaekul et al. (2012). Samples of dried apple snacks were stored at 4°C and were analyzed after an interval of 0, 7, 14, 21, and 28 days. Shortly, all the samples were diluted with deionized water and spread on MRS medium. The Petri plates were incubated at 37°C for a duration of 48 h. The viable cell count was calculated. MRS media and glassware used for viability assessment of probiotics were completely sterilized using the autoclave and hot air oven. After preparation of media, pouring was done in sterilized Petri plates. After complete dilution, the samples were transferred to Petri plates with the help of micro‐dispenser and Petri dishes were incubated for growth.

### 2.12 Sensory analysis

Sensory analysis was performed by following the procedure of Ranadheera et al. (2012) and Amagwula et al. (2022). The sensory panel included 30 experts (15 female, 15 male) within 20–40 years of age. Participants were asked to evaluate the product (dried apple snack) based on overall liking, color, flavor, mouthfeel, and texture using a 9‐point hedonic scale: 1 = dislike extremely and 9 = like extremely.

### 2.13 Statistical analysis

Results from all the technological and physicochemical characteristics of the encapsulated microgels and dried apple snacks were taken in triplicate. All the collected data were expressed as mean ± SD. ANOVA was applied to all the collected data using Statistix10.

## 3 RESULTS AND DISCUSSIONS

### 3.1 Particle size determination

Two different types of materials CaCO_3_ and Mg(OH)_2_ were used along with sodium alginate and whey protein isolate (WPI). Probiotic bacteria were entrapped in these solutions and their particle size was determined as shown in Table 1. The particle size of SA + CaCO_3_ antacid was observed to be the greatest (621 mm) while the particle size of SA + Mg (OH)_2_ was 618 mm. However, the particle size of WPI + Mg (OH)_2_ and WPI+ CaCO_3_ was 550 mm and 543 mm, respectively. From the data, it is evident that the size of CaCO_3_ loaded microgels was greater than the Mg (OH)_2_ microgels. This may be because the solution containing CaCO_3_ had a greater viscosity than the Mg (OH)_2_ solution. The same reasons were also suggested by Smidsrød and Skja (1990). Similar studies have also been reported. Awuchi et al. (2019) reported particle size of grains that can be used for microencapsulation.

**TABLE 1:** Size of prepared beads (mean ± STD)

Beads	Size (μm)
SA + Mg (OH)_2_	618 ± 0.03
SA + CaCO_3_	621 ± 0.14
WPI + Mg (OH)_2_	550 ± 0.08
WPI + CaCO_3_	543 ± 1.11

### 3.2 Encapsulation efficiency

The mean results obtained for encapsulation efficiency of *L. acidophilus* is shown in Table 2. From the results, it can be observed that the microgels with SA + CaCO_3_ showed the highest encapsulation efficiency (95.92%) while WPI + CaCO_3_ was 89.43% efficient. However, the encapsulation efficiency of SA + Mg (OH)_2_ was 86.27% while WPI + Mg(OH)_2_ showed 93.72% efficiency. It was observed that the particle size was influenced by the temperature, viscosity, and concentration of the polymers used along with the encapsulating procedures (Krasaekoopt et al., 2003).

**TABLE 2:** Encapsulation efficiency

Beads	Initial count (before encapsulation) (Log CFU/g)	Final count (after encapsulation) (Log CFU/g)	% Efficiency
SA + Mg (OH)_2_	9.54 ± 0.27	8.23 ± 0.01	86.27
SA + CaCO_3_	9.56 ± 0.21	9.17 ± 0.06	95.92
WPI + Mg (OH)_2_	9.55 ± 0.34	8.95 ± 0.02	93.72
WPI + CaCO_3_	9.55 ± 0.28	8.54 ± 0.01	89.43

### 3.3 Scanning electron microscopy (SEM)

**FIGURE 1:**
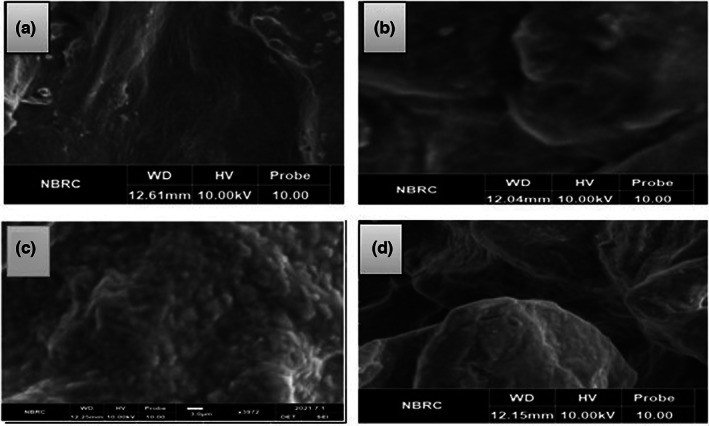
Scanning electron micrographs (a) SA + Mg(OH)_2_ (Sodium alginate microgels with Mg(OH)_2_), (b) SA + CaCO_3_ (Sodium alginate microgels with CaCO_3_), (c) WPI + Mg(OH)_2_ (whey protein isolate microgels with Mg(OH)_2_), (d) WPI + CaCO_3_ (whey protein isolate microgels with CaCO_3_)

The detailed microscopy of the microbeads was carried out using SEM. Obtained micrographs are shown in Figure 1. Micrographs showed that antacid‐containing microbeads have an irregular appearance because of antacid particles present in it. The results of the study are in line with the findings of Min Gu et al. (2019) who observed irregular structure in the case of microgels containing antacid. Probiotics were detected in both types of encapsulated microbeads having Mg (OH)_2_ and CaCO_3_ antacids.

### 3.4 Viability of free and encapsulated probiotics under simulated digestion

**FIGURE 2:**
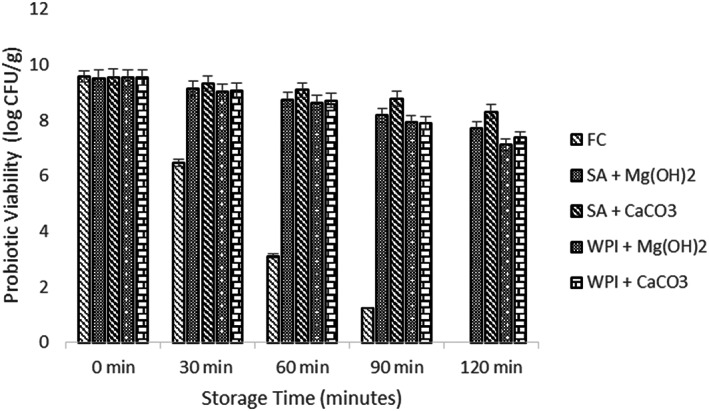
Viability of free and encapsulated (SA and WPI having Mg (OH)_2_ and CaCO_3_ antacids) probiotic microgels under simulated gastric conditions during storage intervals (0, 30, 60, 90, and 120 min) compared with control. Each bar represents the mean value for viability of treatments. F_c_ (un‐encapsulated probiotics), SA + Mg (OH)_2_ (Sodium alginate microgels with Mg (OH)_2_), SA + CaCO_3_ (Sodium alginate microgels with CaCO_3_), WPI + Mg (OH)_2_ (whey protein isolate microgels with Mg (OH)_2_), and WPI + CaCO_3_ (whey protein isolate microgels with CaCO_3_).

**FIGURE 3:**
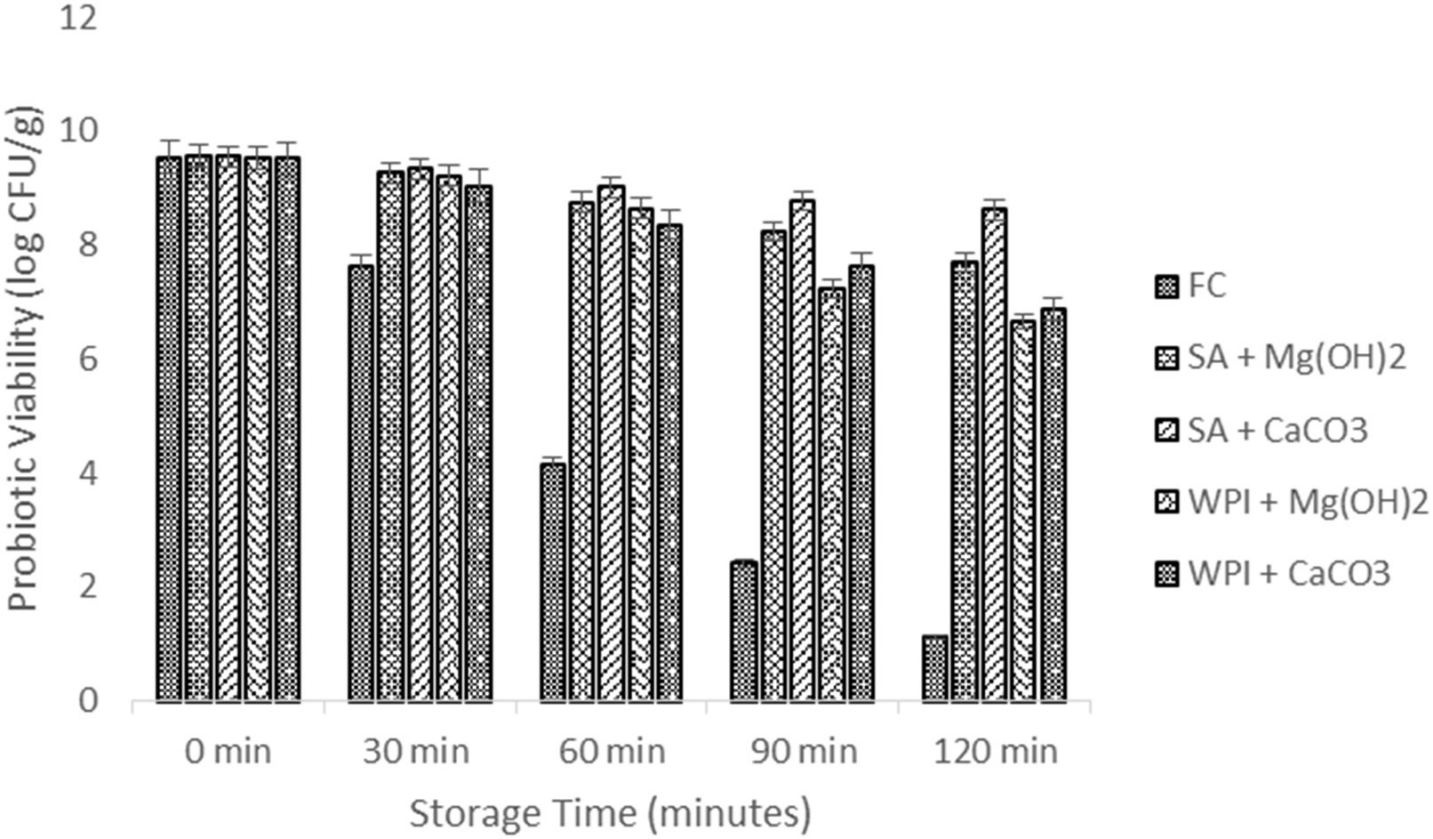
Viability of free and encapsulated (SA and WPI having Mg (OH)_2_ and CaCO_3_ antacids) probiotic microgels under simulated intestinal conditions during storage intervals (0, 30, 60, 90, and 120 min) compared with control. Each bar represents the mean value for viability of treatments. F_c_ (un‐encapsulated probiotics), SA + Mg (OH)_2_ (Sodium alginate microgels with Mg (OH)_2_), SA + CaCO_3_ (Sodium alginate microgels with CaCO_3_), WPI + Mg (OH)_2_ (whey protein isolate microgels with Mg (OH)_2_) and WPI + CaCO_3_ (whey protein isolate microgels with CaCO_3_).

The probiotic load is reduced to a great extent while passing through the gastro‐intestinal environment and due to this reason their survival in the human gut becomes more difficult (Nazzaro et al., 2012). Therefore, the effect of probiotic microgels with both antacid Mg (OH)_2_ and CaCO_3_ was exposed to the simulated gastric and intestinal environment and the mean results were obtained. All the results showed a significant declining trend as shown in Figures 2 and 3. When results for simulated gastric conditions were observed, it showed that the treatment SA + CaCO_3_ had the highest viable population of the probiotics (8.31 log CFU) while the control sample F_c_ had the least probiotic survival rate. The WPI antacid microgels, however, showed lower stability than the SA antacid microgels. A log 1.24 CFU/g and log 2.17 CFU/g reduction were noted in the case of Microgels having CaCO_3_ antacid while a log 1.79 CFU and log 2.42 CFU decrease was observed in the case of Mg (OH)_2_ antacids. However, a decline of 3.43 log CFU/g was calculated in the control sample.

The CaCO_3_ performed better than Mg (OH)_2_ due to the reason that the pH level of microgels that contained Mg (OH)_2_ was near to the neutral pH when subjected to gastric incubation. For this reason, we can also assume that there was enough SA + Mg (OH)_2_ microgel that neutralized the gastric juice and released the probiotics directly in the acidic environment (Zheng et al., 2017). Therefore, microgels that were loaded with CaCO_3_ gave satisfactory results in the gastric environment than the microgels that were loaded with Mg (OH)_2_.

After the simulated gastric environment, viability of probiotic microgels was determined under the simulated intestinal environment. After exposure to the simulated intestinal juice, a sharp fall was observed in the control treatment (F_c_) and a log 4.11 CFU/g fall was noted after 120 min of study. The maximum peak was obtained by SA + CaCO_3_ while other treatment samples showed lower survival results. The difference of the mean results (initial and final) showed 0.93 log CFU/g reduction and 2.63 log CFU/g reduction in the case of SA and WPI with CaCO_3_ antacid. However, 1.87 log CFU/g and 2.88 log CFU/g decline were determined for SA and WPI with Mg (OH)_2_.

The hidden fact involved in the protective properties of both antacids Mg (OH)_2_ and CaCO_3_ is still not clear and will receive further research to reveal the facts. One of the reasons might be that the calcium ions are released at a relatively slower rate and therefore they slowly react with the bile and other digestive salts (Ruiz et al., 2013). Another possible reason could be the size of CaCO_3_ microgels that was greater than Mg (OH)_2_, so it dissolved at a slower rate than Mg (OH)_2_ in aqueous phase (Terpou et al., 2019; Wang et al., 2022).

### 3.5 Thermal resistance

**FIGURE 4:**
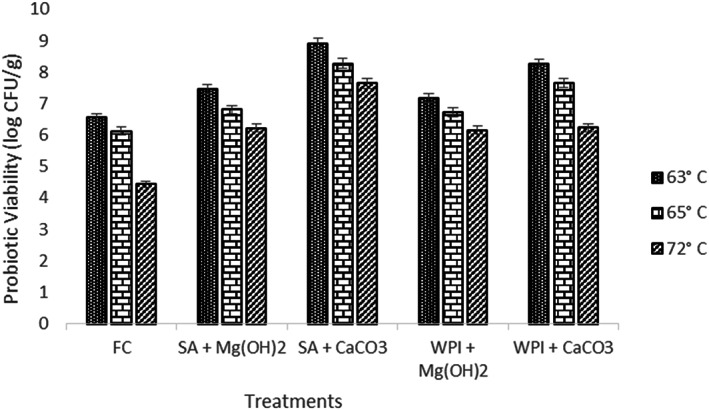
Viability of free and encapsulated (SA and WPI having Mg(OH)_2_ and CaCO_3_ antacids) probiotic microgels at elevated temperature (63, 65, and 72°C) for 10 min compared with control. Each bar represents the mean value for viability of treatments. F_c_ (un‐encapsulated probiotics), SA + Mg (OH)_2_ (Sodium alginate microgels with Mg (OH)_2_), SA + CaCO_3_ (Sodium alginate microgels with CaCO_3_), WPI + Mg (OH)_2_ (whey protein isolate microgels with Mg (OH)_2_), and WPI + CaCO_3_ (whey protein isolate microgels with CaCO_3_).

As *L. acidophilus* can resist heat shocks at higher temperatures (Saarela et al., 2004), it can therefore be subjected to various elevated temperatures. The mean results were obtained against temperature (63, 65, and 72°C). All the results showed a significant decrease in the bacterial population as shown in Figure 4. However, the results from free probiotics (F_c_) showed a sharp decline in viability under elevated temperature.

The decreasing trend in the probiotic population may be due to the reason that high temperatures can cause denaturation and unfolding in the structure of proteins molecules in probiotic cells and can inhibit and denature the enzymatic activity as well which causes the death of live cells of probiotics (Corcoran et al., 2008). Lian et al. (2002) suggested that the wall materials that are used for encapsulation have different physical properties and can act as a barrier against several adverse conditions.

### 3.6 Refrigeration storage

**FIGURE 5:**
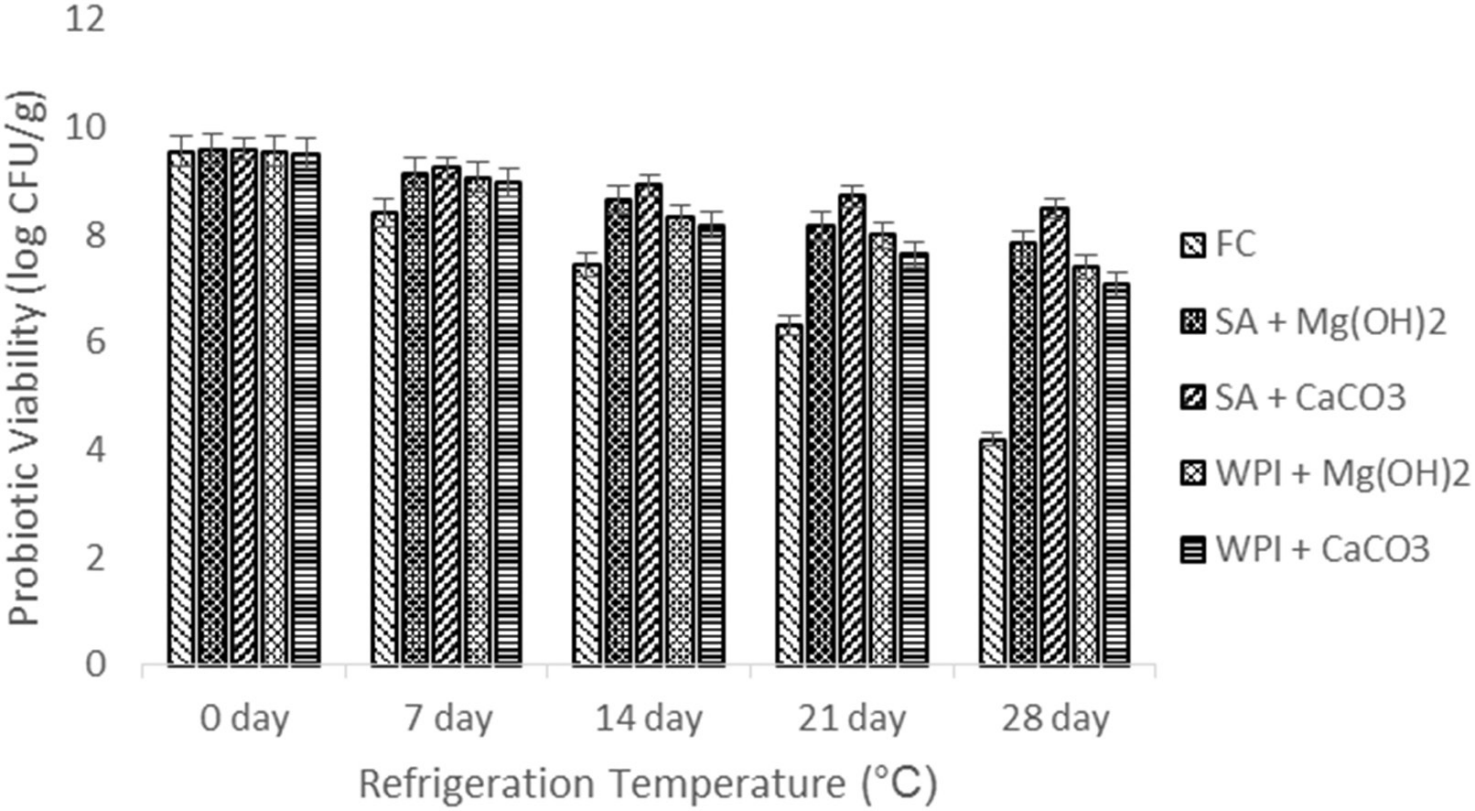
Viability of free and encapsulated (SA and WPI having Mg(OH)_2_ and CaCO_3_ antacids) probiotic microgels during refrigeration storage at 4°C during storage intervals (0, 7, 14, 21, and 28 days) compared with control. Each bar represents the mean value for viability of treatments. F_c_ (un‐encapsulated probiotics), SA + Mg (OH)_2_ (Sodium alginate microgels with Mg (OH)_2_), SA + CaCO_3_ (Sodium alginate microgels with CaCO_3_), WPI + Mg (OH)_2_ (whey protein isolate microgels with Mg (OH)_2_) and WPI + CaCO_3_ (whey protein isolate microgels with CaCO_3_).

Probiotic microgel viabilities (free and encapsulated) were compared during refrigerated storage (4°C) for up to 28 days and mean results are shown in Figure 5. Total cell viability of these samples significantly changed during the storage time; however, the survival of the microgels between 0 day and 7 days suggests that an immediate response to the stress conditions was not induced by the process of encapsulation due to which the viability rate was comparatively higher.

When all mean results were analyzed, the highest viability was exhibited by the hydrogels that were encapsulated in SA + CaCO_3_ antacid solution. However, the results for the same antacid in WPI solution showed the least viability among all the antacid solutions. The control treatment F_c_ showed the viability under acceptable range (10^6^ log CFU).

However, varying degrees of survival of *L. plantarum* were also reported in earlier studies in free and encapsulated form (Coghetto et al., 2016; Trabelsi et al., 2014). Trabelsi et al. (2014) also reported nearly 8 log CFU/ml decline for *L. plantarum* in free form during refrigerated storage over 35 days. However, Brinques and Ayub (2011) reported a reduction of *L. plantarum* BL011 population by half the initial population after about 10 days.

### 3.7 Analysis of dried apple snack

#### 3.7.1 pH

**FIGURE 6:**
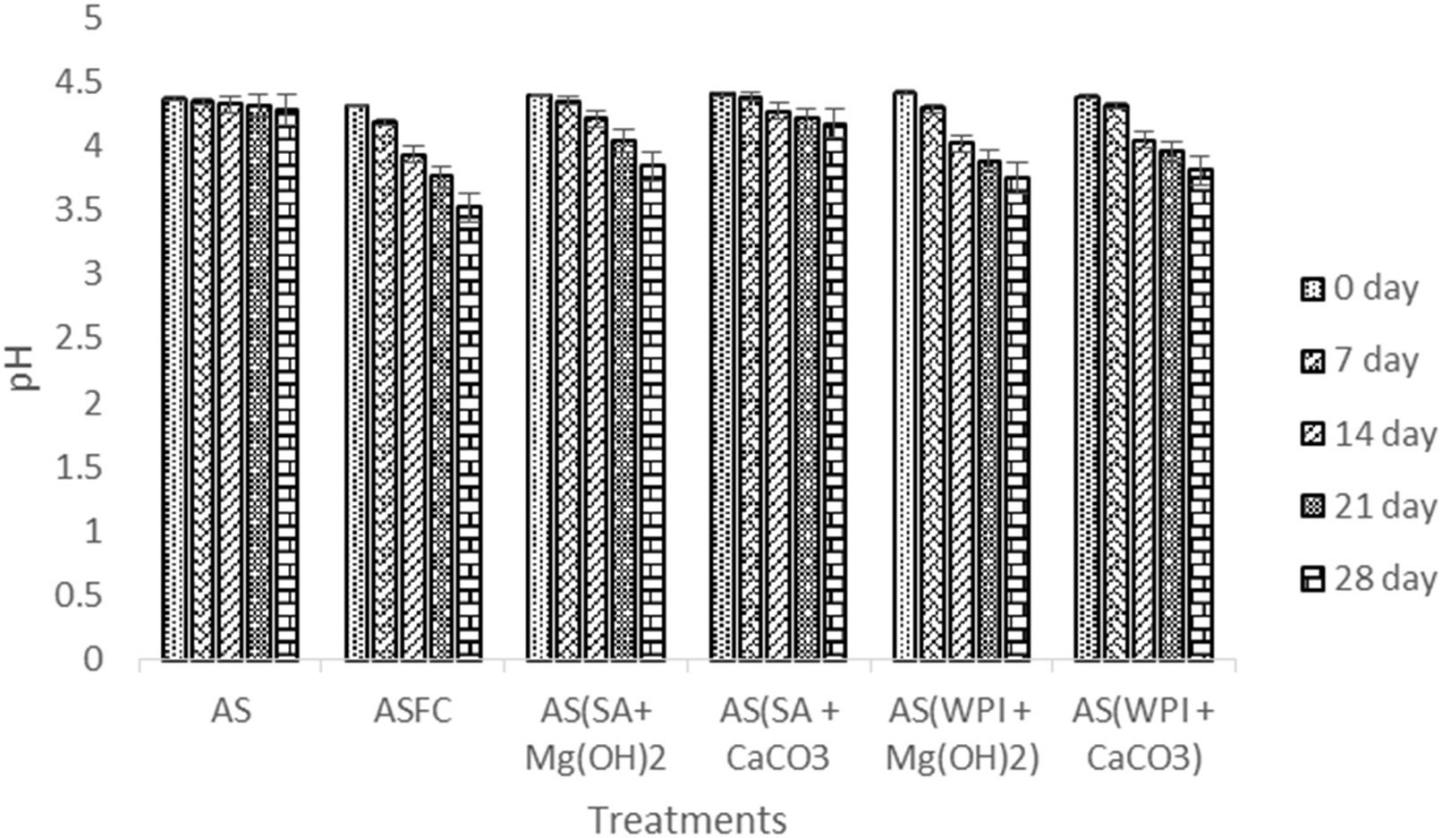
Effect of free (unencapsulated) and encapsulated (SA and WPI having Mg(OH)_2_ and CaCO_3_ antacids) *L. acidophilus* on the pH of dried apple snacks during storage intervals (0, 7, 14, 21, and 28 days) compared with control. Each bar represents the mean value for viability of treatments. AS (control/without probiotics), AS_FC_ (free/unencapsulated cells), AS(SA + Mg(OH)_2_ (apple snack having sodium alginate microgels with Mg(OH)_2_), AS(SA + CaCO_3_) (apple snack having sodium alginate microgels with CaCO_3_), AS(WPI + Mg(OH)_2_) (apple snack having whey protein isolate microgels with Mg(OH)_2_) and AS(WPI + CaCO_3_) (apple snack having whey protein isolate microgels with CaCO_3_).

Dried apple snacks containing microgels were developed and further analyses were carried out to determine the product acceptance as shown in Figure 6. As pH of food is one of the major parameters in determining food quality (Raju et al., 2020), therefore the pH of dried apple snacks impregnated with probiotic microgels was determined. A significant reduction in pH values can be observed. The pH of control treatment (F_c_) did not show a sharp decline; instead, it fell gradually. However, pH of apple snacks having free probiotics was reduced at a faster rate. The maximum pH among microgels was analyzed by AS (SA + CaCO_3_); however, other samples showed lower pH than AS (SA + CaCO_3_). A study on apple snacks was conducted by Mejía‐Águila et al. (2021) and similar pH conditions were reported.

### 3.8 Probiotic viability

**FIGURE 7:**
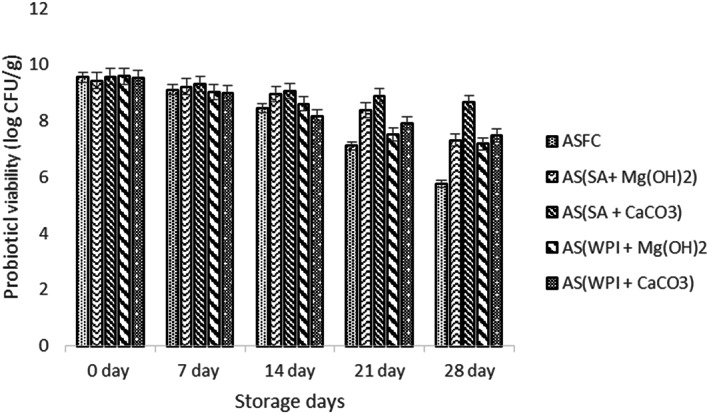
Effect of free (unencapsulated) and encapsulated (SA and WPI having Mg(OH)_2_ and CaCO_3_ antacids) *L. acidophilus* on probiotic viability of dried apple snacks during storage intervals (0, 7, 14, 21, and 28 days) compared with control. Each bar represents the mean value for viability of treatments. AS (control/without probiotics), AS_FC_ (free/unencapsulated cells), AS(SA + Mg(OH)_2_ (apple snack having sodium alginate microgels with Mg(OH)_2_), AS(SA + CaCO_3_) (apple snack having sodium alginate microgels with CaCO_3_), AS(WPI + Mg(OH)_2_) (apple snack having whey protein isolate microgels with Mg(OH)_2_) and AS(WPI + CaCO_3_) (apple snack having whey protein isolate microgels with CaCO_3_).

Mean results for probiotic viability are shown in Figure 7. Overall, a gradual significant decreasing trend was observed. The maximum content of probiotics was observed in apple snacks prepared with *L. acidophilus* encapsulated with alginate and CaCO_3_ (SA + CaCO_3_).

A 0.92 log CFU/g decrease was noted at the end of storage study. However, a log of 2.03 CFU/g was determined in dried apple snacks that contained WPI + CaCO_3_ antacid. Similarly, probiotic viability of the snacks containing Mg (OH)_2_ was observed as log 2.12 CFU/g while log 2.40 CFU/g was detected in apple snack having SA + Mg (OH)_2_ and WPI + Mg (OH)_2_. However, in the case of dried apple snacks having free probiotics (AS_FC_), a piercing drop was monitored in the growth of probiotics. From these results, it can be concluded that microgels containing sodium alginate exhibited better probiotic protection and increased their survival rate. Similar results were also obtained by Afzaal, Saeed, Hussain, et al. (2020). In another study, *Lactobacillus bulgaricus* was encapsulated in wall material and showed enhanced viability. The results of present research are also in accordance with the findings of Li et al. (2018), who observed satisfactory results for *L. plantarum* in apple snacks.

### 3.9 Sensory

**FIGURE 8:**
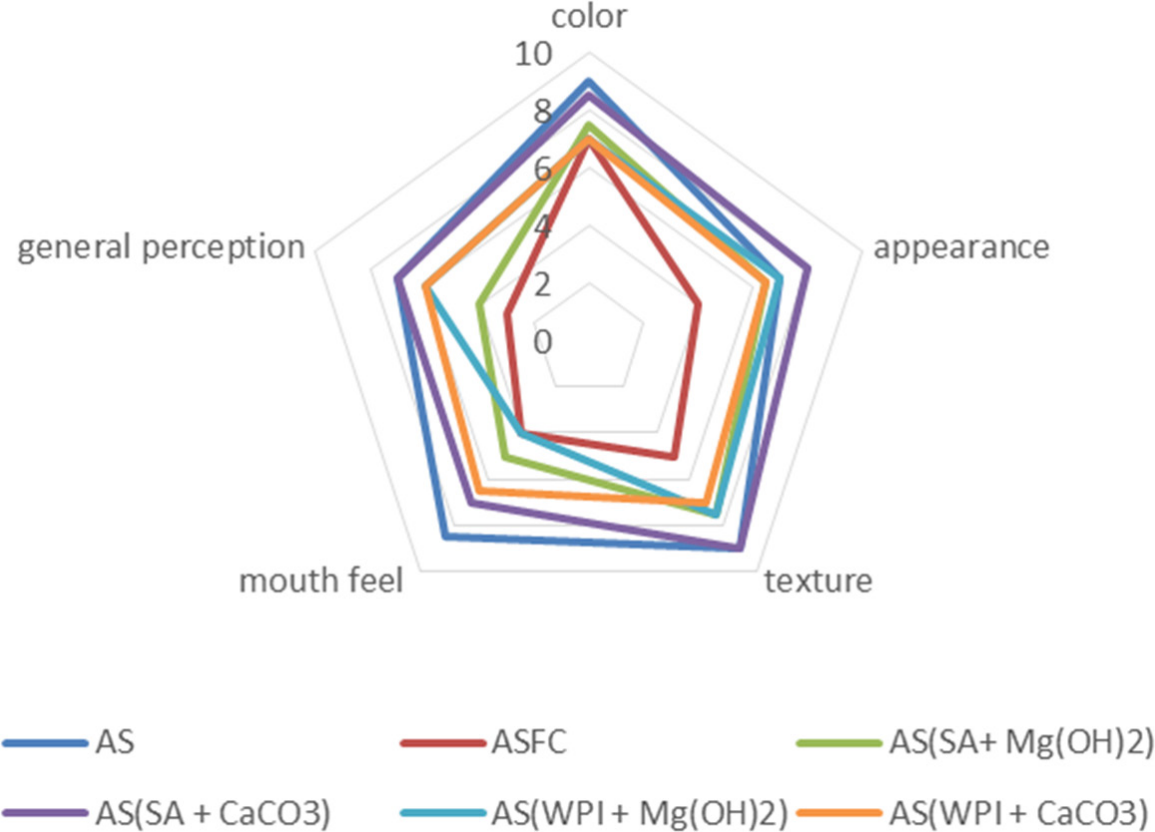
Effect of free (unencapsulated) and encapsulated (SA and WPI having Mg(OH)_2_ and CaCO_3_ antacids) *L. acidophilus* on sensory profile of dried apple snacks during storage intervals (0, 7, 14, 21, and 28 days) compared with control. Each line represents mean value for viability of treatments. AS (control/without probiotics), AS_FC_ (free/unencapsulated cells), AS(SA + Mg(OH)_2_ (apple snack having sodium alginate microgels with Mg(OH)_2_), AS(SA + CaCO_3_) (apple snack having sodium alginate microgels with CaCO_3_), AS(WPI + Mg(OH)_2_) (apple snack having whey protein isolate microgels with Mg(OH)_2_) and AS(WPI + CaCO_3_) (apple snack having whey protein isolate microgels with CaCO_3_).

Sensory results suggested that additions of probiotics affect the sensory profile of any food product. However, the results of the current study revealed that the dried apple snack having microgels can be acceptable by the consumers. The assimilation of probiotic microgels affected significantly (*p* < .05) the sensory parameters (color, texture, appearance, and general perception) of apple snacks as compared to the snack having free probiotic cells. However, the sample (AS_FC_) was not appreciated by the panelists. However, dried apple snack with SA + CaCO_3_ was highly acceptable along with the control sample. A study on dried apple snacks conducted by Afzaal, Saeed, Ateeq, et al. (2020); Afzaal, Saeed, Hussain, et al. (2020); Afzaal, Saeed, Saeed, et al. (2020) also suggested that the wall material incorporated into the apple snack was appreciated by the consumers without changing the sensory profile of the product (Figure 8).

## 4 CONCLUSION

In the present study, microgels loaded with antacids were evaluated for their effect on probiotics viability under stressed conditions. Results indicated that the use of antacids has a key role in sustaining a neutral pH within microgels and ensures safe passage of probiotics through extremely acidic gastric juices and augment the viability of probiotics. Conclusively, CaCO_3_ showed as a more effective antacid agent than Mg (OH)_2_ for protecting the probiotics. Overall, the microgels prepared in this study showed better stability under stress as well as during product storage.

## FUNDING INFORMATION

The authors declare that no funds, grants, or other support were received during the preparation of this manuscript.

## CONFLICT OF INTEREST

Authors declare that they have no conflict of interest.

## ETHICS STATEMENT

This article does not contain any studies with human participants or animals performed by any of the authors.

## CONSENT TO PARTICIPATE

Corresponding and all the co‐authors are willing to participate in this manuscript.

## CONSENT FOR PUBLICATION

All authors are willing for publication of this manuscript.

## Data Availability

Even though adequate data have been given in the form of Tables and Figures, however, all authors declare that if more data are required then the data will be provided on request basis.
